# COMPARISON OF LONELINESS AND SOCIAL NETWORK BETWEEN OLDER ADULT CAREGIVERS AND NONCAREGIVERS

**DOI:** 10.1093/geroni/igad104.0059

**Published:** 2023-12-21

**Authors:** Ting Yong, Abhijit Visaria, Rahul Malhotra

**Affiliations:** Duke-NUS Graduate Medical School, Singapore, Singapore; Duke-NUS Graduate Medical School, Singapore, Singapore; Duke-NUS Graduate Medical School, Singapore, Singapore

## Abstract

In ageing societies, more older adults are taking up informal caregiving roles. Caregivers, compared to non-caregivers, are found to have poorer psychosocial outcomes such as loneliness and social isolation in various studies. However, most of this research is not specific to older adult caregivers and does not account for selection bias into caregiving. We compare loneliness and social network between older adult caregivers and older adult non-caregivers in Singapore, applying inverse probability weighted regression adjustment (IPWRA), to address selection bias into caregiving, on data from a nationally representative longitudinal study of older adults. Those providing unpaid assistance to any person because of their health condition were considered as caregivers. Loneliness (score: 0-12; higher score=lonelier) and social network (0-60; higher score=greater network) were measured using validated scales. The relationship of caregiving status with loneliness and social network was estimated using IPWRA, controlling for multiple demographic and health characteristics. Of the 2639 respondents, 7.2% were caregivers. Older adult caregivers, versus non-caregivers, were more likely to experience loneliness (average treatment effect [ATE] β-coefficient for loneliness score: 0.328 (95% confidence interval: 0.011-0.645; p-value: 0.042); and odds ratio for being lonely: 1.080 (1.001-1.165); p-value: 0.047). However, they did not differ in terms of their social network (ATE β-coefficient for social network score: 1.456 (-0.405-3.316); p-value: 0.125). Our findings suggest that even with a similar extent of social network, older adult caregivers are at a higher risk of loneliness, i.e., perceived social isolation or relational deficits, compared to non-caregivers.

